# Inhibition of prostate cancer growth by solanine requires the suppression of cell cycle proteins and the activation of ROS/P38 signaling pathway

**DOI:** 10.1002/cam4.916

**Published:** 2016-10-10

**Authors:** Bin Pan, Weifeng Zhong, Zhihai Deng, Caiyong Lai, Jing Chu, Genlong Jiao, Junfeng Liu, Qizhao Zhou

**Affiliations:** ^1^ Department of UrologyThe First Affiliated Hospital of Jinan UniversityGuangzhouChina; ^2^ Sun Yat‐sen Cancer CenterState Key Laboratory of Oncology in South China, Collaborative Innovation Center of Cancer MedicineGuangzhouChina; ^3^ Department of UrologyGao Zhou People's HospitalGaozhouChina; ^4^ Department of UrologyZhuhai People's HospitalZhuhaiChina; ^5^ Department of SurgeryThe First Affiliated Hospital of Jinan UniversityGuangzhouChina; ^6^ Department of UrologyInner Mongolia People's HospitalHohhotChina; ^7^ Department of UrologyThe Third Affiliated Hospital of Southern Medical UniversityGuangzhouChina

**Keywords:** Cell cycle proteins, P38, prostate cancer, reactive oxygen species, solanine

## Abstract

Solanine, a naturally steroidal glycoalkaloid in nightshade (Solanum nigrum Linn.), can inhibit proliferation and induce apoptosis of tumor cells. However, the mechanism of solanine‐suppressing prostate cancer cell growth remains to be elucidated. This study investigates the inhibition mechanism of solanine on cancer development in vivo and in cultured human prostate cancer cell DU145 in vitro. Results show that solanine injection significantly suppresses the tumor cell growth in xenograft athymic nude mice. Solanine regulates the protein levels of cell cycle proteins, including Cyclin D1, Cyclin E1, CDK2, CDK4, CDK6, and P21 in vivo and in vitro. Also, in cultured DU145 cell, solanine significantly inhibits cell growth. Moreover, the administration of NAC, an active oxygen scavenger, markedly reduces solanine‐induced cell death. Blockade of P38 MAPK kinase cannot suppress reactive oxygen species (ROS), but can suppress solanine‐induced cell apoptosis. Also, inhibition of ROS by NAC inactivates P38 pathway. Taken together, the data suggest that inhibition of prostate cancer growth by solanine may be through blocking the expression of cell cycle proteins and inducing apoptosis via ROS and activation of P38 pathway. These findings indicate an attractive therapeutic potential of solanine for suppression of prostate cancer.

## Introduction

Prostate cancer is a relatively common malignant cancer and the second leading cause of cancer‐related deaths among male patients 1. Several effective therapies are applied for the clinical treatment of prostate cancer; including surgery and radiotherapy for patients with early‐stage disease and adjuvant systemic treatments, which are effective for advanced‐stage disease 2. However, the therapies all have different side‐effect profiles and are challenged by the emergence of resistance in tumor cells 3, 4. Therefore, it is of particularly importance to develop novel therapeutic strategies to overcome these challenges.

**Figure 1:**
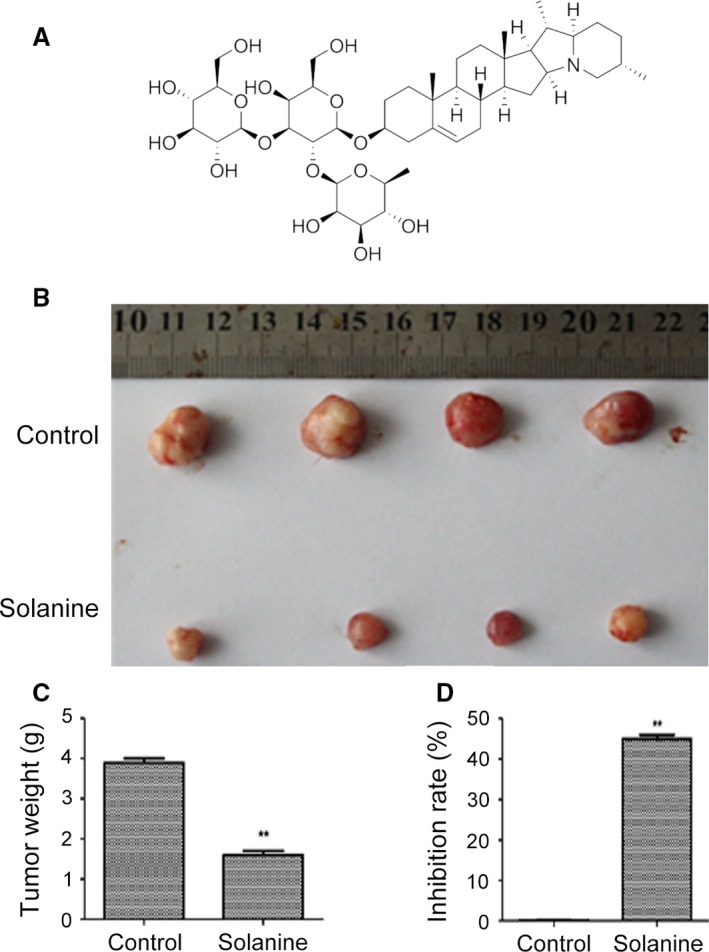
Attenuation of the tumors formed by the inoculation of DU145 cells into nude mice after the treatment with solanine. (A) Chemical formula of solanine. (B) Tumors from the two groups were shown. The statistical data of tumor weight were shown in (C) and the tumor inhibition rate were shown in (D). ** denotes *P *<* *0.01 versus control group.

Solanine, molecular formula C_45_H_73_NO_15_, a trisaccharide glycoalkaloid, is one of the main steroidal glycoalkaloids in Solanaceae family species such as nightshade and potato (*Solanum tuberosum* L.) 5, 6. The structure of solanine (Fig. 1A) is similar to human steroid hormones such as androgen, estrogen, progesterone, and other sex hormones. Solanine, like other alkaloids steroid, has a variety of bio‐activities, such as anti‐inflammatory, preventing asthma, antiallergic, regulate metabolism, and endocrine effects 7. Studies have demonstrated that solanine inhibits the growth of many human cancers, including human colon, liver, cervical, lymphoma, and stomach cancer 8, 9, 10. Solanine also exerts chemoprotective and chemotherapeutic effects in an animal model of breast cancer by induction of apoptosis, and inhibition of cell proliferation and angiogenesis 11. Moreover, solanine inhibits the migration and invasion of human melanoma cells, by reducing matrix metalloproteinase activities 12. Thus, solanine possesses the potential for cancer chemotherapeutic action.

Solanine has been shown anticarcinogenic potential against various cancer cell lines, but the underlying mechanism involved in inhibition of tumor growth by solanine remains to be further elucidated. This work determines the inhibitory effect and the molecular mechanisms of solanine on tumor inhibition in vivo and in vitro. Solanine can inhibit cancer growth in vivo and can induce the apoptosis of prostate cancer cell line DU145, via the suppression of cell cycle protein expression, induction of reactive oxygen species (ROS) and activation of P38 MAPK pathway.

## Methods and Materials

### Ethics statement

All human and animal experiments were approved by the Jinan University. All experiments using human specimens were performed in accordance with the Declaration of Helsinki. Informed consent was received from all participated subjects prior to the study. Human tissue samples were obtained from osteosarcoma patients in the First Affiliated Hospital of Jinan University. All animal procedures followed the humane care guidelines of the Chinese National Institute of Health, and the protocols were approved by the Committee on Animal Research of Jinan University.

### Cell culture

Cell lines human prostate cancer DU145 was purchased from Cell Bank of Shanghai Institute of Cell Biology, Chinese Academy of Sciences. Cells were maintained in Dulbecco's minimal essential medium (DMEM) supplemented with 10% fetal bovine serum (FBS), penicillin (100 U/mL), and streptomycin (100 *μ*g/mL) at 37°C in a humidified atmosphere with 5% CO_2_.

### ROS detection

The intracellular ROS level was detected using an oxidation sensitive fluorescent probe (DCFH‐DA) (Beyotime, China). DCFH‐DA can be deacetylated by nonspecific esterase to form DCFH that can be oxidized by hydrogen peroxide or low‐molecular‐weight peroxides to produce the fluorescent compound 2′,7′‐dichlorofluorescein (DCF) and it is a stable fluorescent ROS‐sensitive compound and can readily diffuse into cells. In this study, DU145 cells were incubated with solanine. The cells were harvested and washed with serum‐free medium for three times. Aliquots of cells were resuspended in fresh medium without serum and loaded with DCFH‐DA (30 *μ*mol/L) for 20 min at 37°C, according to the manufacturer's instruction. Green fluorescence density of 10,000 events was detected by flow cytometry for each sample.

### MTS assay

In vitro DU145 cell proliferation assay was performed using CellTiter 96^®^ AQueous One Solution Cell Proliferation Assay Kit (Promega, Madison, WI), according to the manufacturer's instructions. In brief, 2500 cells per well were seeded into a 96‐well plate. After respective treatments, cells were allowed to grow for 24 h. After 24 h, 20 *μ*L of CellTiter 96 AQueous One Solution reagent was added to each well in 100 *μ*L of total volume of media. Cells were incubated for 3–4 h, and absorbance was recorded at 490 nm using an ELISA plate reader. The absorbance is directly proportional to the number of living cells and is expressed as a relative percentage to vehicle‐treated cells.

### Western blotting

As described previously 13, cells were lysed in sample buffer and subjected to sodium dodecyl sulfate–polyacrylamide gel (SDS‐PAGE), with the antibodies Cyclin D1, Cyclin E1, CDK2, CDK4, CDK6, P21, P38, p‐P38, ATF2, p‐ATF2, Bcl‐2, Bax, and GAPDH (all were from Abcam, Cambridge, UK). GAPDH was used as loading control. After washing with phosphate‐buffered saline (PBS) containing 0.1% Tween 20 for five times, the membrane was incubated the appropriate horseradish peroxidase conjugated secondary antibodies (Amersham Biosciences, Uppsala, Sweden) for 1 h and bands were detected by enhanced chemiluminescence (Amersham, Bucks, UK). In all cases, the average intensity of the pixels in a background‐selected region was calculated and subtracted from each pixel in the sample. To correct for deviation, the densitometry values obtained in the linear range of detection were normalized to those obtained for GAPDH. Statistical analysis was performed using a one‐way analysis of variance (ANOVA), followed by the Bonferroni correction.

### Total RNA extraction and real‐time PCR

Total RNA was isolated using Trizol reagent (Invitrogen, Carlsbad, CA) according to the manufacturer's protocol. For real‐time quantative PCR, equal amount of cDNA was added to SYBR premix EX Taq II (Takara, Japan) and run in Stepone real‐time PCR system (AB Applied Biosystems, Foster City, CA). The cycling program was 95°C for 5 sec and 60°C for 30 sec. Each sample was assayed in triplicates, and GAPDH was used as an endogenous control. Results were normalization to the expression of GAPDH.

### Apoptosis detection by flow cytometry

Cell death via apoptosis was determined using Annexin V‐APC/7‐AAD Apoptosis Kit (BD Biosciences, San Diego, CA) and quantified by flow cytometry as described previously 14, 15. Briefly, at the end of the treatment time**,** DU145 cells were washed once with PBS and harvested in a 0.5% trypsin/EDTA solution at 37°C, centrifuged at 220*g* for 5 min and then immediately resuspended in the physiological buffer provided in the kit. Cells (1 × 10^5^ cells/500 *μ*L) were then maintained in the dark for 15 min at room temperature with both Annexin V, allophycocyanin conjugate (APC) and 7‐amino‐actinomycin D (7‐AAD) accordingly, after which the samples were analyzed immediately by BD Biosciences *FACSCalibur* flow cytometer (San Jose, CA). The results were quantified using the CellQuest software (BD Biosciences, San Jose, CA). Q2 plus Q4 area were calculated as apoptosis ratio.

### Prostate tumor xenograft model

Six‐ to eight‐week‐old male nude mice were kept on a 12 h light–dark cycle with access to food and water ad libitum. Nude mice with subcutaneous DU145 prostate cancer cell xenografts (1 × 10^6^ DU145 cells) were subjected to daily i.p. injections of solanine (5 mg/kg) 16 or vehicle for 4 weeks. After 30 days the mice were killed by ketamin injection (50 mg/kg) followed by cervical dislocation and the xenografts were dissected out and immediately snap frozen in liquid nitrogen and stored at −80°C until further processing.

### Statistical analysis

All experiments were repeated at least three times, and the results are presented as the mean ± SEM. Analyses of significance were performed using Student's *t*‐tests or one‐way ANOVAs, followed by Bonferroni corrections. *P *<* *0.05 was considered statistically significant.

## Results

### Solanine inhibits prostate cancer growth in vivo

To confirm the antitumor effect of solanine in vivo, we performed a xenograft assay of DU145 cell in athymic nude mice. After inoculated human prostate cancer DU145 cells into nude mice subcutaneously, solanine was simultaneously injected into the mice. Afterward, the same amount of solanine was injected to mice every 3 days. One month later, mice‐bearing tumors were killed and the tumors were separated. As shown in Figure 1B, tumors in solanine‐treated mice were much smaller than that in the control mice. The tumor weight was shown in Figure 1C, and the inhibition rate was shown in Figure 1D. These data indicate that solanine significantly inhibit the tumor growth in vivo.

### Solanine regulates the expression of cell cycle proteins in vivo

**Figure 2:**
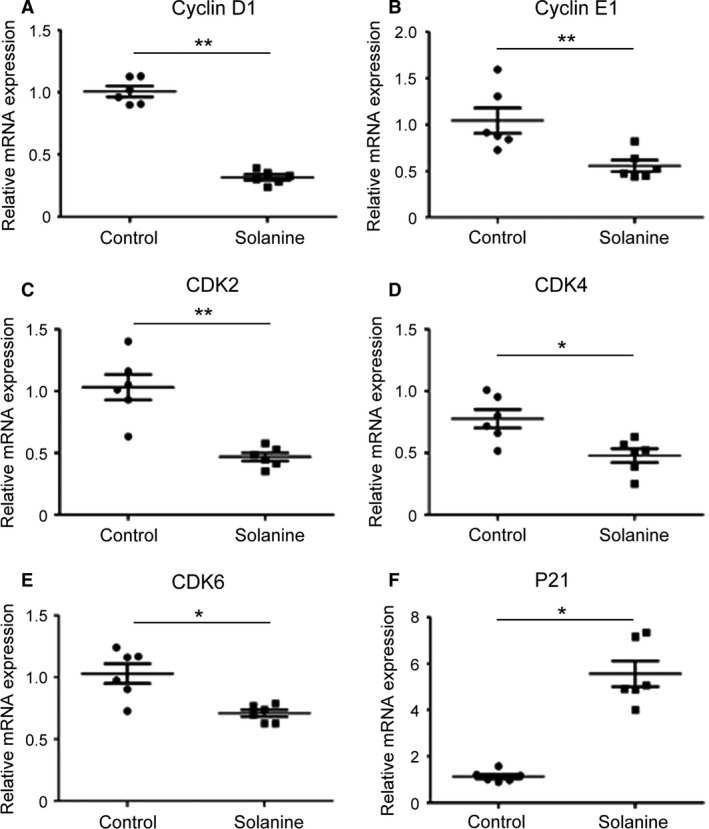
The mRNA levels in xenograft mice injected with DU145 cells. After taken the photos of these tumors in figure 1, the tissues were subjected to QPCR. The mRNA levels of Cyclin D1 (A), Cyclin E1 (B), CDK2 (C), CDK4 (D), CDK6 (E) and P21 (F) in control group were normalized to 1 (100%), the levels in solanine group were reset to relative mRNA expression to control (fold changes). * denotes *P *<* *0.05 versus control group; ** denotes *P *<* *0.01 versus control group.

**Figure 3:**
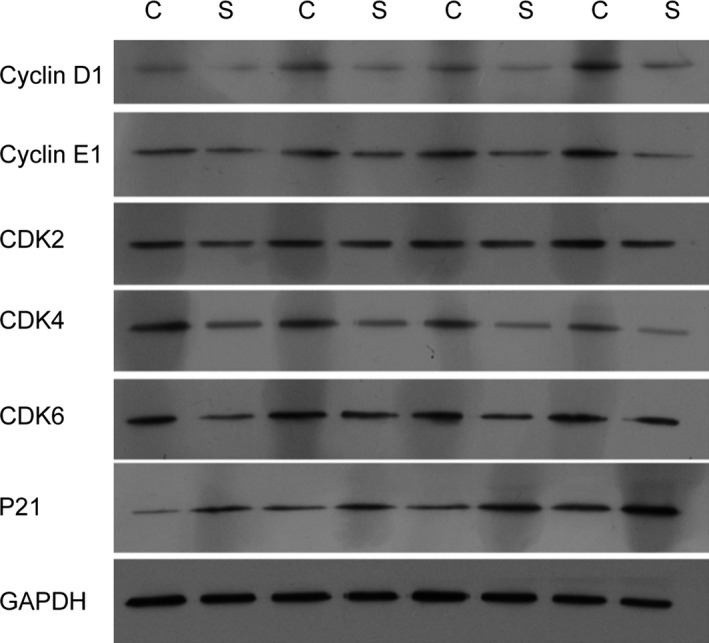
The protein levels in xenograft mice injected with DU145 cells. One month after DU145 cell injection, the tumor were separated and subjected to western blot with the indicated antibodies. C denotes the control group; S denotes the solanine group.

To investigate the mechanisms of solanine regulated tumor inhibition, we detected the mRNA and protein expression levels of cell cycle proteins from the tumor tissues of xenograft mice injected with DU145 cells. As shown in Figure 2, by the performance of QPCR, we found that compared to control group, the mRNA levels of CyclinD1, CyclinE1, CDK2, CDK4, and CDK6 were significantly inhibited and the level of P21 was markedly increased. Furthermore, we performed western blotting and found the same trend as the mRNA. Compared to control group, the protein levels of CyclinD1, CyclinE1, CDK2, CDK4, and CDK6 were significantly decreased and that of P21 was increased (Fig. 3).

### Solanine inhibits the cell growth of prostate cancer cell DU145

**Figure 4:**
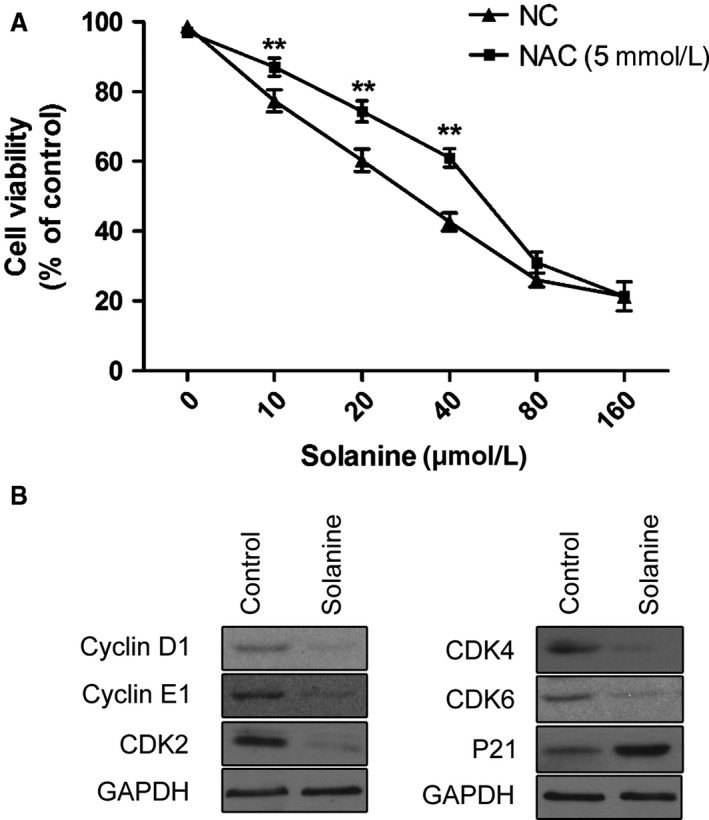
Solanine inhibit DU145 cell growth via ROS. (A) Different concentrations (10–160 *μ*mol/L) of solanine with or without NAC (5 mmol/L), were applied on cultured DU145. After the treatment, cells were harvested and subjected to MTS. The cell viability was calculated under different treatment group. ** denotes *P *<* *0.01 versus NC (control for NAC) group. (B) Cultured DU145 treated with solanine or control were subjected to western blot with the indicated antibodies. ROS, reactive oxygen species.

To explore the detailed mechanisms and signaling pathways of solanine‐induced cell growth, we moved our study to cultured DU145 cells in vitro. First, we need to confirm the appropriate concentration of solanine on DU145 cells, by MTS assay. Concentrations of 10–160 *μ*mol/L solanine were used on DU145 cells. At 24 h, the calculated IC50 of solanine was 32.18 *μ*mol/L, so the concentration of 40 *μ*mol/L solanine can significantly inhibit the cell growth. We chose this concentration for our further study. Meanwhile, we used an active oxygen scavenger NAC (5 mM) to inhibit ROS and found that the usage of NAC significantly reversed the inhibition effect of solanine, leading to the increased use of concentration of solanine to suppress the cell growth. Concentrations lower than 80 *μ*mol/L showed remarkable effect (Fig. 4A). These data indicated that solanine inhibited prostate cancer DU145 cell via ROS. Moreover, we determined the protein levels of CyclinD1, CyclinE1, CDK2, CDK4, CDK6, and p21 in DU145 cells. And found the same trend as that in tumor tissues of xenograft mice (Fig. 4B), indicating the consistence of mechanisms of solanine‐induced cell death in vivo and in vitro.

### Solanine induces ROS in prostate cancer cell DU145

**Figure 5:**
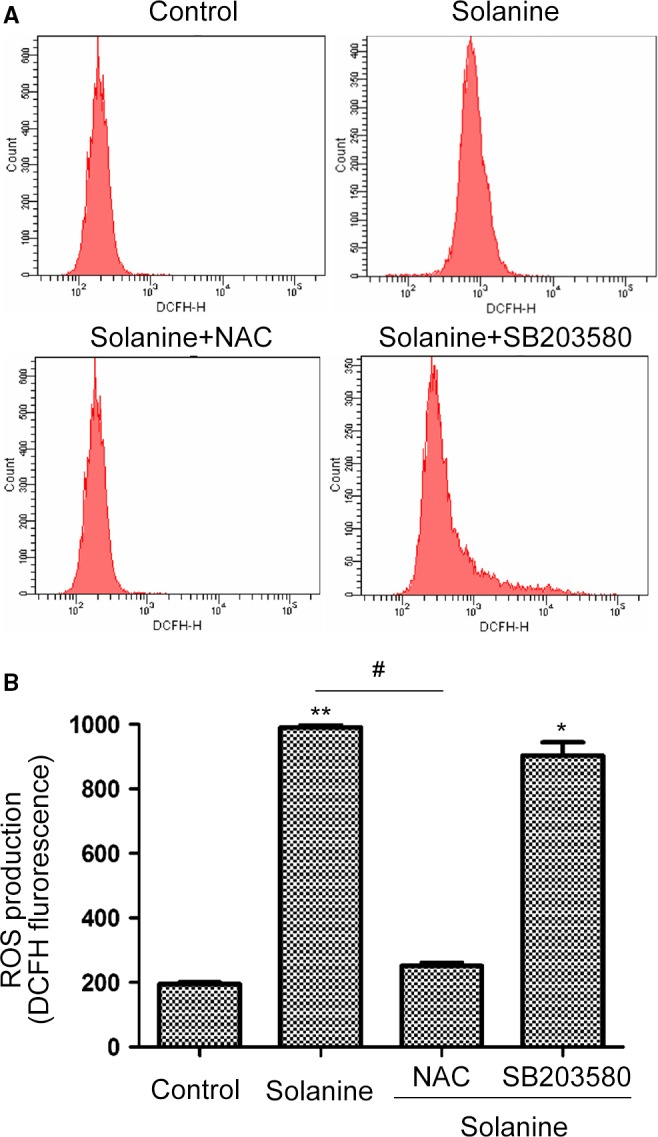
Solanine induces reactive oxygen species (ROS) in DU145 cells. (A) Cells were treated solanine with or without NAC (5 mmol/L) or SB203580 (40 *μ*mol/L) for 24 h. Then cells were subjected to DCFH staining for the detection of ROS by flow cytometry. (B) The statistical data were shown. * denotes *P *<* *0.05 versus control group; ** denotes *P *<* *0.01 versus control group; # denotes *P *<* *0.05 versus solanine group.

To further confirm the relationship of solanine and ROS, we applied flow cytometry assay to detect the ROS level under different treatment, using an oxidation‐sensitive fluorescent probe (DCFH‐DA) 17. As shown in Figure 5, the treatment of solanine significantly induced ROS, and the usage of NAC markedly erased solanine‐induced ROS level. Meanwhile, we used SB203580, an inhibitor of P38 MAPK kinase pathway, together with solanine. We found that SB203580 cannot suppress the ROS level induced by solanine. These data confirmed that solanine‐induced DU145 ROS and this cannot be inhibited by P38 inhibitor.

### Solanine induces apoptosis in DU145 cells via P38 pathway downstream of ROS

**Figure 6:**
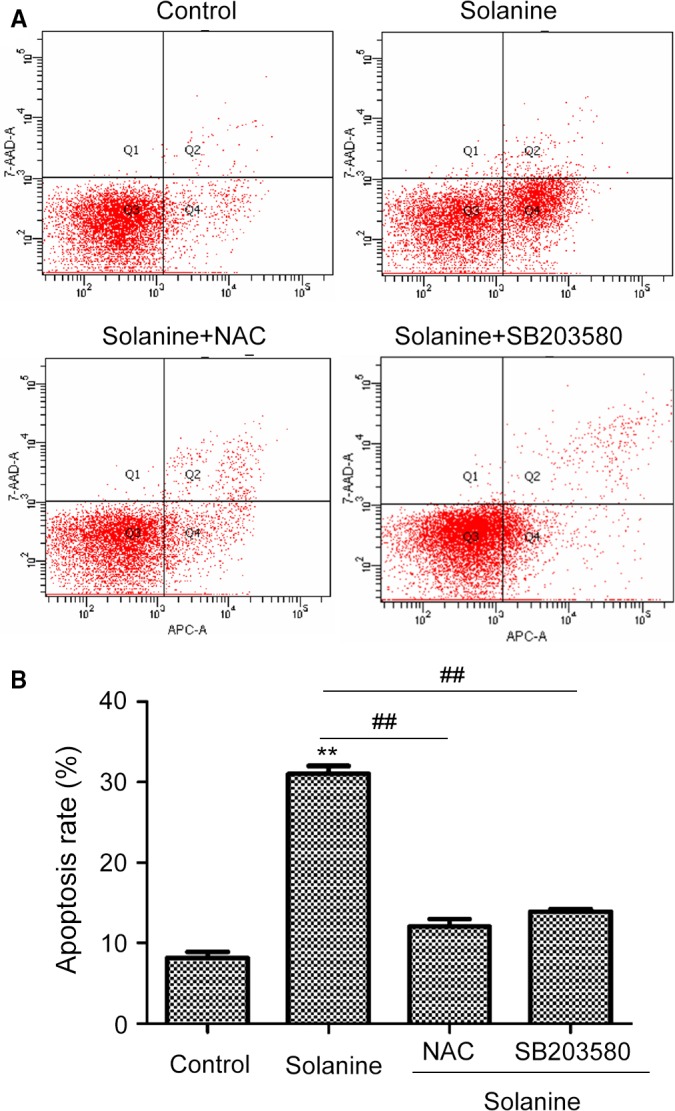
Cell apoptosis of DU145 detected by flow cytometry. (A) DU145 cells treated as the same as in Figure 5 were stained with Annexin V and 7‐AAD, then cells were subjected to flow cytometry to detect cell apoptosis. The representative scatter plots were shown in (A) and the statistical data were shown in (B). ** denotes *P *<* *0.01 versus control group; ## denotes *P *<* *0.01 versus solanine group.

**Figure 7:**
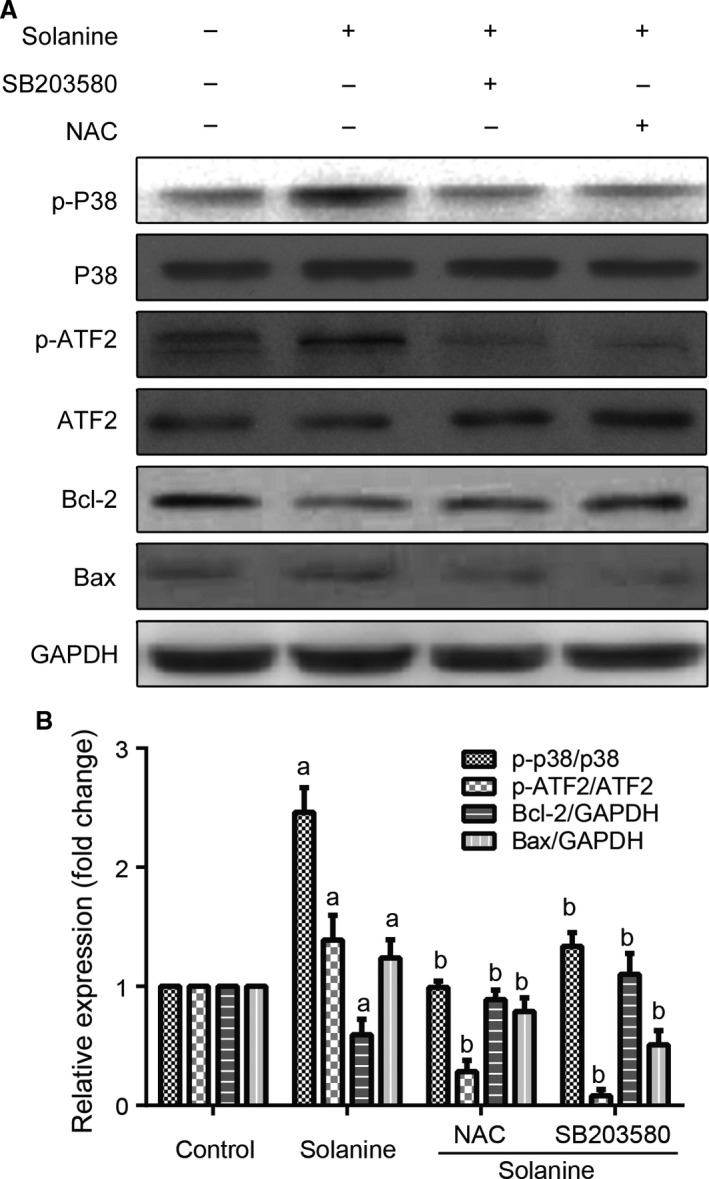
P38 activation detected by western blot in DU145 cells. DU145 cells were treated with solanine, together with or without NAC (5 mmol/L) or SB203580 (40 *μ*mol/L) as in Figure 5. The cells were harvested and subjected to western blot with indicated antibodies. The representative scatter plots were shown in (A) and the statistical data were shown in (B). a denotes *P *<* *0.05 versus control group; b denotes *P *<* *0.05 versus solanine group.

After demonstrated solanine can inhibit prostate cancer growth and induce ROS in cultured DU145, we further need to know which signaling pathway was responsible. So we performed flow cytometry to detect the cell apoptosis. By the staining of Annexin V‐APC and 7‐AAD with flow cytometry, we found that solanine significantly induced the apoptosis of DU145 cells, the administration of NAC and SB203580 can markedly decrease solanine‐induced cell apoptosis (Fig. 6). Furthermore, we need to explore the signaling mechanisms underlying. By western blot, we detected the protein expression and activation of P38 pathway. As shown in Fig. 7, solanine induced the expression of phosphorylated P38, not the total level of P38, indicating the activation of P38 pathway. ATF2 is a downstream target of P38 18. We found the protein level of phosphorylated ATF2 was also increased, but not the total level. The administration of NAC and SB203580 both can suppress solanine‐induced P38/ATF2 activation. Furthermore, we determined the levels of pro and antiapoptotic proteins. As shown in Fig. 7, the level of antiapoptotic protein Bcl‐2 was decreased and the level of proapoptotic protein Bax was increased, and these proteins responded with NAC and P38 inhibitor. These data suggest that solanine induces cell apoptosis via P38 pathway, downstream of ROS induction.

## Discussion

In recent years, the incidence of prostate cancer was significantly increased. Advanced primary treatment of prostate cancer is endocrine therapy, which would lead to hormonal resistance 19, 20. Thus, researches on new treatments and new drugs for prostate cancer have important significance. Solanine, an important glycoalkaloid in nightshade, exhibits anticarcinogenic potentials, inhibiting cell growth of various cancer cell lines and inducing apoptosis of human cancer cells 8, 9, 10. The present findings demonstrate that solanine suppresses tumor growth of DU145 in xenograft athymic nude mice in vivo and in cultured DU145 cell in vitro. Solanine regulates the mRNA and protein levels of many cell cycle proteins, induces cell apoptosis via the induction of ROS and the activation of P38 MAPK pathway, downstream of ROS.

The activation of CDKs (cyclin‐dependent kinases) is critical for cells entering S phase from G1, which are regulated by the periodic expression of cyclins. Cyclin D/E forms complexes with and activates CDK 2, 4, or 6, promoting cell transition from G1 to S phase 21, 22. P21, a CDK inhibitor (CKDI), regulates the negative feedback of G1/S transition, and increased expression of P21 blocks the progress of G1/S transition 23, 24. Our study of qPCR and western blots show that solanine administration can significantly downregulate the expression of Cyclin D1/E1, CDK 4 and 6, upregulate P21, indicating that solanine affects the G1/S transition signal, blocks the progress of G1/S transition, inhibits cell growth of cancer cell and tumor growth in vivo. The detailed mechanisms of how solanine influences the signal of cell cycle G1/S transition needs to be further explored. Staurosporine can cause the increasing of Ca^2+^ and accumulation of ROS, and induce cell apoptosis 25. So, solanine may induce ROS accumulation to damage DNA, upregulate the expression of P21, restrain the expression of c‐Myc, thus block cell G1/S progress.

ROS is important for the development and metastasis 26. Recent studies show that regulating ROS can reach the therapeutic purposes by accelerating tumor cell death 27, 28. The level of ROS in cancer cells is high, so cancer cells are always under oxidative stress, leading to the higher sensitivity than healthy cells. This provides theoretical basis for the application of pro‐oxidant drugs, such as trioxide, gemcitabine, for curing cancers 29, 30, 31, 32. ROS suppresses cancer development mainly through the following aspects: (1) promoting cell apoptosis, (2) inducing the necrosis and (3) regulating autophagic cell death 33, 34. Our data show the solanine induces ROS. Once the active oxygen scavenger NAC is used, solanine‐induced cell growth inhibition and apoptosis are all suppressed, indicating ROS plays an important role solanine‐regulated apoptosis.

P38, a MAPK kinase protein, is an important regulator of inflammation, which can be activated by physiological stress, lipopolysaccharide, osmotic stress, and UV irradiation 35, 36, 37. Lipopolysaccharide, tumor necrosis factor, platelet‐activating factor, IL‐1, and other inflammatory stimuli can induce the activation of P38 in endogenous immune cells such as monocytes, endothelial cells, and neutrophils 38, 39. P38 activation is closely related to apoptosis and P38 can increase the expression of c‐Myc, phosphorylation of P53 and activation of c‐Jun, to regulate apoptosis 40, 41. ATF2 is a commonly recognized downstream target of P38 MAPK kinase 18. P38 phosphorylates Thr‐69 and Thr‐71 of ATF2, leading to the activation of ATF2. Activated ATF2 forms dimer with c‐Jun to activate caspase‐3, thus leading the mitochondrial‐dependent apoptosis 42. We found the downregulation of Bcl‐2 and upregulation of Bax when treated with solanine. The unbalance of Bcl‐2/Bax leads to the mitochondrial membrane permeabilization of mitochondria, leading to the release of cytochrome C, and finally irreversible apoptosis 43. Moreover, our results show that after the treatment of solanine in DU145 cells, the expression level of phosphorylated P38/ATF2 is significantly upregulated, which can be blocked by the administration of P38 inhibitor SB203580 and the active oxygen scavenger NAC, indicating the P38 pathway is downstream of ROS induction in solanine‐induced apoptosis and tumor inhibition.

In summary, the data in this study demonstrates the solanine, through the suppression of cell cyclin proteins to inhibit cancer development and through the induction of ROS, promotes P38 activation to induce DU145 cell apoptosis. This data elucidates the mechanisms of solanine‐induced prostate cancer cell inhibition, providing theoretical and experimental foundation for the clinical application of solanine.

## Conflict of Interest

The authors declare no conflicts of interest.

## References

[1] Jemal, A. , R. Siegel , J. Xu , and E. Ward . 2010 Cancer statistics, 2010. CA Cancer J. Clin. 60:277–300. 20610543 10.3322/caac.20073

[2] Jani, A. B. , and S. Hellman . 2003 Early prostate cancer: clinical decision‐making. Lancet 361:1045–1053. 12660074 10.1016/S0140-6736(03)12833-4

[3] Ganswindt, U. , A. Stenzl , M. Bamberg , and C. Belka . 2008 Adjuvant radiotherapy for patients with locally advanced prostate cancer–a new standard? Eur. Urol. 54:528–542. 18602742 10.1016/j.eururo.2008.06.059

[4] Kent, E. C. , and M. H. Hussain . 2003 Neoadjuvant Therapy for Prostate Cancer: An Oncologist's Perspective. Rev. Urol. 5(Suppl 3):S28–S37. PMC1502344 16985947

[5] Ji, Y. B. , S. Y. Gao , C. F. Ji , and X. Zou . 2008 Induction of apoptosis in HepG2 cells by solanine and Bcl‐2 protein. J. Ethnopharmacol. 115:194–202. 18022776 10.1016/j.jep.2007.09.023

[6] Friedman, M. 2006 Potato glycoalkaloids and metabolites: roles in the plant and in the diet. J. Agric. Food Chem. 54:8655–8681. 17090106 10.1021/jf061471t

[7] Barceloux, D. G. 2009 Potatoes, tomatoes, and solanine toxicity (*Solanum tuberosum* L., *Solanum lycopersicum* L.). Dis. Mon. 55:391–402. 19446683 10.1016/j.disamonth.2009.03.009

[8] Lee, K. R. , N. Kozukue , J. S. Han , J. H. Park , E. Y. Chang , E. J. Baek , et al. 2004 Glycoalkaloids and metabolites inhibit the growth of human colon (HT29) and liver (HepG2) cancer cells. J. Agric. Food Chem. 52:2832–2839. 15137822 10.1021/jf030526d

[9] Friedman, M. , K. R. Lee , H. J. Kim , I. S. Lee , and N. Kozukue . 2005 Anticarcinogenic effects of glycoalkaloids from potatoes against human cervical, liver, lymphoma, and stomach cancer cells. J. Agric. Food Chem. 53:6162–6169. 16029012 10.1021/jf050620p

[10] Yang, S. A. , S. H. Paek , N. Kozukue , K. R. Lee , and J. A. Kim . 2006 Alpha‐chaconine, a potato glycoalkaloid, induces apoptosis of HT‐29 human colon cancer cells through caspase‐3 activation and inhibition of ERK 1/2 phosphorylation. Food Chem. Toxicol. 44:839–846. 16387404 10.1016/j.fct.2005.11.007

[11] Mohsenikia, M. , A. M. Alizadeh , S. Khodayari , H. Khodayari , S. A. Kouhpayeh , A. Karimi , et al. 2013 The protective and therapeutic effects of alpha‐solanine on mice breast cancer. Eur. J. Pharmacol. 718:1–9. 24051269 10.1016/j.ejphar.2013.09.015

[12] Lu, M. K. , Y. W. Shih , T. T. Chang Chien , L. H. Fang , H. C. Huang , and P. S. Chen . 2010 alpha‐Solanine inhibits human melanoma cell migration and invasion by reducing matrix metalloproteinase‐2/9 activities. Biol. Pharm. Bull. 33:1685–1691. 20930376 10.1248/bpb.33.1685

[13] Wang, L. , X. Y. Li , G. F. Jiang , J. Z. Liang , Y. Sun , and W. Liu . 2013 Reversal effect of BM‐cyclin 1 on multidrug resistance by down‐regulating MRP2 in BALB/C nude mice bearing C‐A120 cells. J. Huazhong Univ. Sci. Technolog. Med. Sci. 33:840–844. 24337845 10.1007/s11596-013-1208-6

[14] Umemura, M. , E. Baljinnyam , S. Feske , M. S. De Lorenzo , L. H. Xie , X. Feng , et al. 2014 Store‐operated Ca^2+^ entry (SOCE) regulates melanoma proliferation and cell migration. PLoS ONE 9:e89292. 24586666 10.1371/journal.pone.0089292 PMC3931742

[15] Kaur, T. , D. Mukherjea , K. Sheehan , S. Jajoo , L. P. Rybak , and V. Ramkumar . 2011 Short interfering RNA against STAT1 attenuates cisplatin‐induced ototoxicity in the rat by suppressing inflammation. Cell Death Dis. 2:e180. 21776018 10.1038/cddis.2011.63 PMC3199718

[16] Lv, C. , H. Kong , G. Dong , L. Liu , K. Tong , H. Sun , et al. 2014 Antitumor efficacy of alpha‐solanine against pancreatic cancer in vitro and in vivo. PLoS ONE 9:e87868. 24505326 10.1371/journal.pone.0087868 PMC3914882

[17] Li, J. J. , Q. Tang , Y. Li , B. R. Hu , Z. Y. Ming , Q. Fu , et al. 2006 Role of oxidative stress in the apoptosis of hepatocellular carcinoma induced by combination of arsenic trioxide and ascorbic acid. Acta Pharmacol. Sin. 27:1078–1084. 16867262 10.1111/j.1745-7254.2006.00345.x

[18] Waas, W. F. , H. H. Lo , and K. N. Dalby . 2001 The kinetic mechanism of the dual phosphorylation of the ATF2 transcription factor by p38 mitogen‐activated protein (MAP) kinase alpha. Implications for signal/response profiles of MAP kinase pathways. J. Biol. Chem. 276:5676–5684. 11069918 10.1074/jbc.M008787200

[19] Zhang, H. Y. , J. Cui , Y. Zhang , Z. L. Wang , T. Chong , and Z. M. Wang . 2016 Isoflavones and Prostate Cancer: A Review of Some Critical Issues. Chin. Med. J. (Engl). 129:341–347. 26831238 10.4103/0366-6999.174488 PMC4799580

[20] Lei, J. H. , L. R. Liu , Q. Wei , T. R. Song , L. Yang , Y. Meng , et al. 2016 Androgen‐deprivation therapy alone versus combined with radiation therapy or chemotherapy for nonlocalized prostate cancer: a systematic review and meta‐analysis. Asian J. Androl. 18:102–107. 25851657 10.4103/1008-682X.150840 PMC4736336

[21] Liao, Y. , Y. Feng , J. Shen , F. J. Hornicek , and Z. Duan . 2016 The roles and therapeutic potential of cyclin‐dependent kinases (CDKs) in sarcoma. Cancer Metastasis Rev. 35:151–163. 26669603 10.1007/s10555-015-9601-1

[22] Simmons Kovacs, L. A. , D. A. Orlando , and S. B. Haase . 2008 Transcription networks and cyclin/CDKs: the yin and yang of cell cycle oscillators. Cell Cycle 7:2626–2629. 18758238 10.4161/cc.7.17.6515

[23] Perkins, N. D. 2002 Not just a CDK inhibitor: regulation of transcription by p21(WAF1/CIP1/SDI1). Cell Cycle 1:39–41. 12429907

[24] Ball, K. L. 1997 p21: structure and functions associated with cyclin‐CDK binding. Prog. Cell. Cycle. Res. 3:125–134. 9552411 10.1007/978-1-4615-5371-7_10

[25] Gil, J. , S. Almeida , C. R. Oliveira , and A. C. Rego . 2003 Cytosolic and mitochondrial ROS in staurosporine‐induced retinal cell apoptosis. Free Radic. Biol. Med. 35:1500–1514. 14642398 10.1016/j.freeradbiomed.2003.08.022

[26] Nishikawa, M. , M. Hashida , and Y. Takakura . 2009 Catalase delivery for inhibiting ROS‐mediated tissue injury and tumor metastasis. Adv. Drug Deliv. Rev. 61:319–326. 19385054 10.1016/j.addr.2009.01.001

[27] Subramani, R. , E. Gonzalez , A. Arumugam , S. Nandy , V. Gonzalez , J. Medel , et al. 2016 Nimbolide inhibits pancreatic cancer growth and metastasis through ROS‐mediated apoptosis and inhibition of epithelial‐to‐mesenchymal transition. Sci. Rep. 6:19819. 26804739 10.1038/srep19819 PMC4726267

[28] Qin, W. , C. Li , W. Zheng , Q. Guo , Y. Zhang , M. Kang , et al. 2015 Inhibition of autophagy promotes metastasis and glycolysis by inducing ROS in gastric cancer cells. Oncotarget 6:39839–39854. 26497999 10.18632/oncotarget.5674 PMC4741864

[29] You, B. R. , and W. H. Park . 2012 Arsenic trioxide induces human pulmonary fibroblast cell death via increasing ROS levels and GSH depletion. Oncol. Rep. 28:749–757. 22684917 10.3892/or.2012.1852

[30] Park, W. H. 2012 MAPK inhibitors and siRNAs differentially affect cell death and ROS levels in arsenic trioxide‐treated human pulmonary fibroblast cells. Oncol. Rep. 27:1611–1618. 22293863 10.3892/or.2012.1661

[31] Wu, W. , Q. Xia , R. J. Luo , Z. Q. Lin , and P. Xue . 2015 In vitro Study of the Antagonistic Effect of Low‐dose Liquiritigenin on Gemcitabine‐induced Capillary Leak Syndrome in Pancreatic Adenocarcinoma via Inhibiting ROS‐ Mediated Signalling Pathways. Asian Pac. J. Cancer Prev. 16:4369–4376. 26028101 10.7314/apjcp.2015.16.10.4369

[32] Fiorini, C. , M. Cordani , G. Gotte , D. Picone , and M. Donadelli . 2015 Onconase induces autophagy sensitizing pancreatic cancer cells to gemcitabine and activates Akt/mTOR pathway in a ROS‐dependent manner. Biochim. Biophys. Acta 1853:549–560. 25533084 10.1016/j.bbamcr.2014.12.016

[33] Li, Z. Y. , Y. Yang , M. Ming , and B. Liu . 2011 Mitochondrial ROS generation for regulation of autophagic pathways in cancer. Biochem. Biophys. Res. Commun. 414:5–8. 21951851 10.1016/j.bbrc.2011.09.046

[34] Simon, H. U. , A. Haj‐Yehia , and F. Levi‐Schaffer . 2000 Role of reactive oxygen species (ROS) in apoptosis induction. Apoptosis 5:415–418. 11256882 10.1023/a:1009616228304

[35] Woll, S. , R. Windoffer , and R. E. Leube . 2007 p38 MAPK‐dependent shaping of the keratin cytoskeleton in cultured cells. J. Cell Biol. 177:795–807. 17535969 10.1083/jcb.200703174 PMC2064280

[36] Cheng, H. , J. Kartenbeck , K. Kabsch , X. Mao , M. Marques , and A. Alonso . 2002 Stress kinase p38 mediates EGFR transactivation by hyperosmolar concentrations of sorbitol. J. Cell. Physiol. 192:234–243. 12115730 10.1002/jcp.10134

[37] Chen, W. , and G. T. Bowden . 1999 Activation of p38 MAP kinase and ERK are required for ultraviolet‐B induced c‐fos gene expression in human keratinocytes. Oncogene 18:7469–7476. 10602506 10.1038/sj.onc.1203210

[38] Arana‐Argaez, V. E. , V. Delgado‐Rizo , O. E. Pizano‐Martinez , E. A. Martinez‐Garcia , B. T. Martin‐Marquez , A. Munoz‐Gomez , et al. 2010 Inhibitors of MAPK pathway ERK1/2 or p38 prevent the IL‐1{beta}‐induced up‐regulation of SRP72 autoantigen in Jurkat cells. J. Biol. Chem. 285:32824–32833. 20729213 10.1074/jbc.M110.121087 PMC2963399

[39] Moretti, M. , J. Budni , A. E. Freitas , V. B. Neis , C. M. Ribeiro , G. de Oliveira Balen , et al. 2015 TNF‐alpha‐induced depressive‐like phenotype and p38(MAPK) activation are abolished by ascorbic acid treatment. Eur. Neuropsychopharmacol. 25:902–912. 25836357 10.1016/j.euroneuro.2015.03.006

[40] Li, F. X. , L. Z. Huang , C. Dong , J. P. Wang , H. J. Wu , and S. M. Shuang . 2015 Down‐regulation of aquaporin3 expression by lipopolysaccharide via p38/c‐Jun N‐terminal kinase signalling pathway in HT‐29 human colon epithelial cells. World J. Gastroenterol. 21:4547–4554. 25914463 10.3748/wjg.v21.i15.4547 PMC4402301

[41] Liu, B. , B. Yuan , L. Zhang , W. Mu , and C. Wang . 2015 ROS/p38/p53/Puma signaling pathway is involved in emodin‐induced apoptosis of human colorectal cancer cells. Int J Clin Exp Med. 8:15413–15422. 26629030 PMC4658919

[42] Song, B. , B. Xie , C. Wang , and M. Li . 2011 Caspase‐3 is a target gene of c‐Jun:ATF2 heterodimers during apoptosis induced by activity deprivation in cerebellar granule neurons. Neurosci. Lett. 505:76–81. 21996423 10.1016/j.neulet.2011.09.060

[43] Kroemer, G. , L. Galluzzi , and C. Brenner . 2007 Mitochondrial membrane permeabilization in cell death. Physiol. Rev. 87:99–163. 17237344 10.1152/physrev.00013.2006

